# Analyses of mitochondrial genes reveal two sympatric but genetically divergent lineages of *Rhipicephalus appendiculatus* in Kenya

**DOI:** 10.1186/s13071-016-1631-1

**Published:** 2016-06-22

**Authors:** Esther G. Kanduma, Joram M. Mwacharo, Naftaly W. Githaka, Peter W. Kinyanjui, Joyce N. Njuguna, Lucy M. Kamau, Edward Kariuki, Stephen Mwaura, Robert A. Skilton, Richard P. Bishop

**Affiliations:** Biosciences eastern and central Africa - International Livestock Research Institute (BecA-ILRI) Hub, P.O. Box 30709-00100, Nairobi, Kenya; Present Address: Department of Biochemistry, School of Medicine, University of Nairobi, P.O. Box 30197-00100, Nairobi, Kenya; Centre for Genetics and Genomics, School of Life Sciences, University Park, University of Nottingham, Nottingham, NG7 2RD UK; International Centre for Agricultural Research in the Dry Areas (ICARDA), P.O. Box 5689, Addis Ababa, Ethiopia; International Livestock Research Institute (ILRI), P.O. Box 30709-00100, Nairobi, Kenya; Department of Zoological Sciences, Kenyatta University, P.O Box 43844-00100, Nairobi, Kenya; Kenya Wildlife Service (KWS), P.O. Box 40241-00100, Nairobi, Kenya; Present Address: International Centre of Insect Physiology and Ecology (icipe), P.O. Box 30772-00100, Nairobi, Kenya

**Keywords:** Ticks, COI, 12S rRNA, ITS2, Phylogeography, Population genetics, Genetic differentiation, Genetic markers, East coast fever

## Abstract

**Background:**

The ixodid tick *Rhipicephalus appendiculatus* transmits the apicomplexan protozoan parasite *Theileria parva,* which causes East coast fever (ECF), the most economically important cattle disease in eastern and southern Africa. Recent analysis of micro- and minisatellite markers showed an absence of geographical and host-associated genetic sub-structuring amongst field populations of *R. appendiculatus* in Kenya. To assess further the phylogenetic relationships between field and laboratory *R. appendiculatus* tick isolates, this study examined sequence variations at two mitochondrial genes, cytochrome *c* oxidase subunit I (COI) and 12S ribosomal RNA (rRNA), and the nuclear encoded ribosomal internal transcribed spacer 2 (ITS2) of the rRNA gene, respectively.

**Results:**

The analysis of 332 COI sequences revealed 30 polymorphic sites, which defined 28 haplotypes that were separated into two distinct haplogroups (A and B). Inclusion of previously published haplotypes in our analysis revealed a high degree of phylogenetic complexity never reported before in haplogroup A. Neither haplogroup however, showed any clustering pattern related to either the geographical sampling location, the type of tick sampled (laboratory stocks *vs* field populations) or the mammalian host species. This finding was supported by the results obtained from the analysis of 12S rDNA sequences. Analysis of molecular variance (AMOVA) indicated that 90.8 % of the total genetic variation was explained by the two haplogroups, providing further support for their genetic divergence. These results were, however, not replicated by the nuclear transcribed ITS2 sequences likely because of recombination between the nuclear genomes maintaining a high level of genetic sequence conservation.

**Conclusions:**

COI and 12S rDNA are better markers than ITS2 for studying intraspecific diversity. Based on these genes, two major genetic groups of *R. appendiculatus* that have gone through a demographic expansion exist in Kenya. The two groups show no phylogeographic structure or correlation with the type of host species from which the ticks were collected, nor to the evolutionary and breeding history of the species. The two lineages may have a wide geographic distribution range in eastern and southern Africa. The findings of this study may have implications for the spread and control of *R. appendiculatus*, and indirectly, on the transmission dynamics of ECF.

**Electronic supplementary material:**

The online version of this article (doi:10.1186/s13071-016-1631-1) contains supplementary material, which is available to authorized users.

## Background

Knowledge relating to the intra- and inter-population genetic structure and variability amongst parasitic populations is important in understanding the dispersal and transmission dynamics of the pathogens they transmit. Several factors, including climate, host diversity, degree of tolerance of host species and control and management practices affecting host behavior are all thought to influence spatial distribution patterns of ticks [1]. The interaction between ticks and their hosts could result in genetic adaptations and divergence that may ultimately lead to genetic differentiation and speciation in ticks. The host’s physiological, behavioral and demographic variability may also influence the genetic landscape of ectoparasites with limited dispersal ability such as ticks [2, 3]. Other factors that are thought to influence the genetic variability of ticks include host availability and migration, ecological requirements of juvenile and adult stages, and tick dispersal ability [4]. For instance, different vertebrate hosts have been shown to influence the genetic structure of *Ixodes uriae* [5], while the availability of suitable hosts to the juvenile stages of *Hyalomma rufipes* and *Amblyomma hebraeum* can influence the geographical distribution of the adult stages of these two ixodid ticks [6].

*Rhipicephalus appendiculatus* is a three-host tick species whose ability to survive in a particular locality is determined by climatic conditions [7, 8] and it almost entirely depends on its hosts for dispersal. It is widely distributed in eastern, central and southern Africa [9, 10]. It lays eggs off its hosts and uses more than one host at different life-cycle stages, specifically larval, nymphal and adult instars. Large numbers of both adult and immature ticks can be found on cattle, goats, African buffalo (*Syncerus caffer*), Waterbuck (*Kobus ellipsiprymnus*), Eland (*Taurotragus oryx*), Greater kudu (*Tragelaphus strepsiceros*) and other large bovids [9]. The larval and nymphal stages frequently infest lagomorphs e.g. the Cape hare (*Lepus capensis*). *Rhipicephalus appendiculatus* is of major economic importance as the vector of the protozoan parasite *Theileria parva*, which causes East coast fever (ECF) in cattle [11]. *Rhipicephalus appendiculatus* also transmits *Theileria taurotragi* to cattle from Eland (*Taurotragus oryx*) causing benign bovine theileriosis, *Anaplasma marginale* resulting in bovine anaplasmosis, the nairovirus inducing Nairobi sheep disease, and *Rickettsia conorii* resulting in tick typhus in humans [9]). Heavy infestations can lead to tick worry, damaged hides - especially the ears where *R. appendiculatus* often congregate, anemia and toxicosis that results in enhanced susceptibility to other diseases [12].

Several studies suggest that phenotypic diversity exists between different populations of *R. appendiculatus*. These include diapause in *R. appendiculatus* in southern Africa, which has not been observed in east African populations [13], differences in body size [10, 14], vector competence [15] and in response to acaricides [16]. Morphological, physiological, epidemiological and phylogenetic data have shown the existence of two groups of *R. appendiculatus* in southern and eastern Africa, which were thought to represent two phylogeographically differentiated lineages [13, 17–19]. Differences in agro-ecological and climatic conditions were thought to drive the differentiation of the two lineages [17–20]. A recent analysis of micro- and minisatellite markers showed an absence of geographic and host-associated genetic structuring amongst field populations of *R. appendiculatus* in Kenya [21].

Several populations of *R. appendiculatus* have been maintained as laboratory stocks for sporozoite production and as representatives of field genotypes. For example, the standard laboratory stock of *R. appendiculatus* (designated Muguga) has been used to produce the Muguga cocktail vaccine against *T. parva* [22, 23]. Previously, analysis of the biology of laboratory stocks of *R. appendiculatus* revealed differences in infection rates [24], and susceptibility to - and efficiency of acquisition of *T. parva* [15, 25]. Recent assessments using micro- and minisatellite markers revealed distinct genetic groups in laboratory stocks of *R. appendiculatus* which were less diverse than their field counterparts [21]. Selection, reproductive isolation and inbreeding were thought to have led to the differentiation in the laboratory stocks. However, this finding has not been investigated further using genetic markers targeting the mitochondrial genome.

While the distribution of *R. appendiculatus* in Africa is determined by ecoclimatic factors, the genetic variability within the species remains poorly investigated. To further assess the phylogenetic relationships between field and laboratory *R. appendiculatus* tick stocks, this study examined sequence variation at the cytochrome *c* oxidase subunit I (COI) gene, 12S rDNA and the bi-parentally inherited ribosomal nuclear ITS2 region. The phylogenetic relationships, demographic dynamics and the partition of genetic diversity and structure amongst populations of *R. appendiculatus* were investigated.

## Methods

### Tick samples

The study used tick samples that had previously been described in earlier studies on population genetics of *R. appendiculatus* [21, 26]. Genomic DNA from a total of 332 individuals from ten field populations and 12 laboratory maintained stocks of *R. appendiculatus* were used to sequence the mitochondrial COI gene. From the 332 samples, a subset of 93 samples from 12 populations was used to sequence the 12S rRNA gene while 87 ticks from the same subset were used to sequence the nuclear ITS2 gene spacer (Additional file 1: Table S1). These samples were randomly selected to represent tick populations falling within the two major COI haplogroups observed in this study. Of the ten field populations, six (118 individuals) came from areas grazed exclusively by cattle, two (43 individuals) from areas grazed exclusively by wildlife, and another two (46 individuals) came from areas co-grazed by wildlife and cattle. A total of 125 individuals were sampled from 12 laboratory colonies, which had been bred and maintained as closed genetic stocks (see [27, 28]). One laboratory stock was originally sampled in Uganda (*n* = 12), one in Zimbabwe (West Mashonaland; *n* = 12) and two in Zambia (Eastern Province; *n* = 12; Southern Province; *n* = 8); the remaining eight stocks were collected in Kenya. The ticks had been identified following standard morphological criteria [29–31]. Details of the area of origin of the ticks, population and sampling site characteristics and the population codes used are as previously described in Kanduma et al. [26]. A list of all the study populations is given in Table 1.

**Table 1:** Summary of COI sequence variability and genetic diversity measures of 22 *R. appendiculatus* populations

Populationª	Sample size	No. of haplotypes	Haplotype diversity (SD)	Nucleotide diversity ± (SD)	Mean number of nucleotide differences (SD)	Fu’s *F*_*S*_ (*P*-value)	Tajima’s D (*P*-value)	Sum of squared deviation (SSD) (*P*-value)	Harpending’s Raggedness Index (RI)	GenBank Accession numbers
Field (Cattle only)										
Kilifi (KF)	20	2	0.100 (0.088)	0.0002 (0.0002)	0.100 (0.1775)	-0.879 (0.080)	−1.164 (0.123)	0.000 (0.288)	0.650 (0.810)	KX276888–89
Makuyu (MK)	25	9	0.817 (0.055)	0.005 (0.001)	2.820 (1.539)	-1.340 (0.264)	−1.015 (0.175)	0.029 (0.140)	0.080 (0.228)	KX276901–09
Kitale (KT)	29	10	0.865 (0.037)	0.010 (0.006)	5.584 (2.760)	0.747 (0.678)	0.999 (0.860)	0.039 (0.489)	0.030 (0.747)	KX276890–99
Busia (BU)	18	7	0.784 (0.085)	0.011 (0.006)	6.183 (3.0824)	2.062 (0.8440)	0.463 (0.715)	0.089 (0.210)	0.099 (0.421)	KX276868–74
Rusinga (RU)	21	5	0.700 (0.073)	0.004 (0.002)	2.010 (1.178)	0.781 (0.690)	−1.749 (0.026)	0.128 (0.180)	0.096 (0.280)	KX276934–38
Ruma (RUM2)	5	4	0.900 (0.161)	0.0145 (0.004)	8.100 (4.534)	1.261 (0.657)	1.497 (0.932)	0.120 (0.131)	0.130 (0.839)	KX276930–33
Average	20	20	0.833 (0.0238)	0.0127 (0.007)	7.089 (3. 352)	0.423 (0.611)	1.477 (0.934)	0.072 (0.030)	0.0479 (0.060)	
Field (Cattle – Wildlife only)										
Field Ol Pejeta (FP)	23	10	0.830 (0.067)	0.005 (0.0017)	2.988 (1.620)	-2.258 (0.109)	−1.536 (0.048)	0.004 (0.792)	0.015 (0.983)	KX276875–84
Bomet (BO)	23	6	0.719 (0.074)	0.010 (0.0024)	5.518 (2.7532)	3.628 (0.936)	0.479 (0.7160)	0.100 (0.168)	0.227 (0.090)	KX276862–67
Average	23	14	0.792 (0.056)	0.008 (0.0043)	4.315 (2.175)	-1.578 (0.322)	−0.576 (0.297)	0.025 (0.370)	0.039 (0.650)	
Field (Wildlife only)										
Nairobi National Park (NB)	21	6	0.729 (0.065)	0.00450 (0.002)	2.5095 (1.408)	0.468 (0.640)	−1.600 (0.038)	0.0246 (0.279)	0.079 (0.521)	KX276924–29
Maasai Mara (MA)	22	6	0.788 (0.050)	0.01022 (0.002)	5.7013 (2.839)	3.618 (0.925)	1.086 (0.889)	0.0691 (0.304)	0.0761 (0.556)	KX276910–15
Average	22	7	0.801 (0.031)	0.091 (0.005)	5.102 (2.518)	4.639 (0.957)	1.294 (0.924)	0.439 (0.470)	0.034 (0.820)	
Laboratory stocks										
Lab Ol Pejeta (LP)	14	1	0 (0)	0 (0)	0 (0)	0	0.000 (1.000)	0.0000	0.00 (0.000)	KX276900
Kiambu High-line (KH)	12	2	0.485 (0.106)	0.0104 (0.0023)	5.8182 (2.993)	9.2418 (0.998)	1.9499 (0.984)	0.4701 (0.0000)	0.7355 (0.924)	KX276885–86
Kiambu unselected line (KU)	10	1	0 (0)	0 (0)	0 (0)	0	0.000 (1.000)	0.0000	0.000 (0.000)	KX276887
Muguga infected (MF)^b^	12	3	0.545 (0.144)	0.0116 (0.003)	6.4849 (3.300)	6.887 (0.995)	1.701 (0.978)	0.4418 (0.000)	0.449 (0.957)	KX276916–18
Muguga unselected (MU)^c^	12	4	0.682 (0.102)	0.0092 (0.003)	5.1364 (2.677)	3.752 (0.953)	0.1463 (0.570)	0.1545 (0.103)	0.3693 (0.700)	KX276919–22
Muguga low-line (ML)^d^	11	1	0 (0)	0 (0)	0 (0)	0	0.000 (1.000)	0.0000	0.000 (0.000)	KX276923
Uganda (UG)	12	1	0 (0)	0 (0)	0 (0)	0	0.000 (1.000)	0.0000	0.000 (0.000)	KX276941
South Africa Natal province (SAN)	5	1	0 (0)	0 (0)	0 (0)	0	0.000 (1.000)	0.0000	0.000 (0.000)	KX276940
South Africa Lab stock (SAL)	5	1	0 (0)	0 (0)	0 (0)	0	0.000 (1.000)	0.0000	0.000 (0.000)	KX276939
Zambia Sothern province (ZS)	8	1	0 (0)	0 (0)	0 (0)	0	0.000 (1.000)	0.0000	0.000 (0.000)	KX276943
Zambia Eastern province (ZE)	12	1	0 (0)	0 (0)	0 (0)	0	0.000 (1.000)	0.0000	0.000 (0.000)	KX276942
Zimbabwe West Mashonaland (ZM)	12	1	0 (0)	0 (0)	0 (0)	0	0.000 (1.000)	0.0000	0.000 (0.000)	KX276944
Average (Lab stocks)	10	5	0.634 (0.023)	0.012 (0.006)	6. 445 (3.022)	15.042 (0.994)	3.198 (0.996)	0.190 (0.040)	0.297 (0.130)	
Haplogroups										
Haplogroup A	193	20	0.664 (0.035)	0.003 (0.002)	1.569 (0.939)	-10.348 (0.00)	−1.650 (0.017)	0.0126 (0.550)	0.050 (0.740)	
Haplogroup B	139	8	0.514 (0.046)	0.001 (0.001)	0.651 (0.507)	-3.462 (0.057)	−1.087 (0.125)	0.0028 (0.170)	0.102 (0.210)	
Overall	332	28	0.802 (0.014)	0.012 (0.006)	6.865 (3.239)	−0.122 (0.549)	1.244 (0.914)	0.076 (0.090)	0.061 (0.070)	

The number of individual sequences analysed (sample size), number of haplotyes and their corresponding GenBank Accession numbers for each population are listed. Tajima’s D was negative and statistically significant in RU, FP and NB but positive and not significant for all the other tick populations except the nine laboratory stocks that did not show diversity. KH had the highest Fu’s FS (9.2418). SSD was statistically significant in KH, MF, RE and RZ

ªPopulation: Source population or origin of the tick stock. Tick populations were grouped on the basis of the source of the sequences as field ticks (collected from areas grazed by cattle), Field (Cattle – Wildlife only) collected from areas co-grazed by cattle and wildlife, laboratory stocks maintained at ILRI Tick Unit and wildlife only ticks (collected from areas grazed by wildlife)

^b^Muguga infected (MF): *T. parva*-infected ticks derived from the original unselected Muguga stock (MU)

^c^This is the laboratory stock used in Kenya for T. parva transmission studies. It was originally collected from the Central Highlands of Kenya in the 1950s and has subsequently been maintained at the East African Veterinary Research Organisation (EAVRO) (now Kenya Agricultural Research Institute (KARI)) and ILRI [27]

^d^Muguga low-line (ML): This selected stock was derived from a family line of Muguga that had low susceptibility to *T. parva* infection [24]

### DNA extraction and PCR amplification

The DNeasy® Blood and Tissue Kit (Qiagen GmbH, Hilden, Germany) was used to extract genomic DNA following minor modifications to the protocol (see [26]). COI gene was amplified using primers described in Folmer et al. [32] while the 12S rRNA gene was amplified using primers described in Simon et al. [33]. The ITS2 region (1–1.25 kb) was PCR amplified as two fragments: a full-length fragment, plus an internal 721 bp fragment to ensure good sequence coverage. The full-length fragment was amplified with the forward primer 3SAF [34] and reverse primer ITS2R [35]. The sequences of the primers used to PCR amplify the COI, 12S rRNA and the nuclear ITS2 fragment and their corresponding annealing temperatures are shown in Additional file 2: Table S2. All PCRs were carried out in 50 μl volumes containing 1X PCR buffer (Promega), 0.125 μmol MgCl_2_, 0.1 μM of each dNTP, 0.25 pmol of each primer, 1.25 U of *Taq* DNA polymerase (Promega) and 50 ng of template DNA. The PCR cycling profiles involved an initial denaturation at 95 °C for 5 min followed by 35 cycles of 94 °C for 1 min, annealing for 1 min (see Additional file 2: Table S2 for annealing temperatures) and extension at 72 °C for 90 s for COI and 2 min for 12S rDNA and ITS2, respectively. A final extension step at 72 °C for 10 min completed the amplification. PCR products were purified using the QIAquick® PCR Purification Kit (Qiagen GmbH, Hilden, Germany) following the manufacturer’s protocol. The products were sequenced directly using the BigDye Terminator v3.1 cycle sequencing chemistry on an ABI 3730 DNA Analyzer in accordance with the manufacturer’s methods (Applied Biosystems, UK).

### Sequence editing and multiple alignments

All sequence chromatograms were visually inspected and the sequences edited manually using the CLC Main Workbench 6.8.3 (CLC bio, Qiagen GmbH, Hilden, Germany). The sequences were then trimmed to remove low quality reads at the 5' and 3' ends. Consensus sequences for each gene were generated from the sequenced fragments. Prior to analyses, all sequences were trimmed to uniform sizes (COI, 558 bp; 12S rDNA, 345 bp; ITS2, 1149 bp). Multiple sequence alignments were performed for each gene using ClustalW2 in CLC Main Workbench. Species identity was investigated and confirmed via BLASTN searches on the NCBI database (http://blast.ncbi.nlm.nih.gov/Blast.cgi).

### Genetic variation and structure

Sequences were collapsed into haplotypes, following multiple sequence alignments, using DnaSP v5.10.01 [36]. Genetic variation represented as nucleotide and haplotype diversity and mean number of nucleotide differences for the COI gene were calculated for each population, groups of populations and haplogroups using DnaSP. The partition of genetic variation within and among populations was assessed via nested analysis of molecular variance (AMOVA) using Arlequin v3.5 [37]. The groupings used in AMOVA were as follows: (i) one group composed of all sequences of *R. appendiculatus*; (ii) two groups of sequences, i.e. those from areas grazed exclusively by cattle *vs* those from areas co-grazed by cattle and wildlife; (iii) two groups of sequences, i.e. those from areas grazed exclusively by cattle *vs* those from areas grazed exclusively by wildlife; (iv) two groups of sequences, i.e. those from areas co-grazed by wildlife and cattle *vs* those from areas grazed exclusively by wildlife; (v) two groups of sequences, i.e. field stocks *vs* laboratory stocks; (vi) three groups of sequences defined on the basis of the host species, i.e. cattle *vs* mixed cattle-wildlife *vs* wildlife, respectively; and (vii) amongst the groups identified by the phylogenetic and median-joining network analysis.

### Demographic dynamics and phylogenetic structure

Demographic dynamics were inferred from mismatch distribution patterns [38–40] of COI haplotypes as implemented in Arlequin. The goodness-of-fit of the observed pattern of mismatches from the one expected under neutrality was tested using the sum of squares deviation (SSD) and Harpending’s raggedness index “RI” [39] following 1000 coalescent simulations. The mismatch distributions were augmented with the Fu’s *F*_S_ [41] and Tajima’s *D* [42, 43] statistics which are also coalescent-based estimators of selective neutrality. Their significance was tested with 1000 coalescent simulations in Arlequin.

Phylogenetic reconstruction was performed using the COI gene employing the Maximum Likelihood (ML) algorithm implemented in MEGA v6.0 [44]. The best nucleotide substitution model for the gene was T92 + G model [45] as determined with MEGA v6.0. Clade support was assessed via 1000 bootstrap replicates. To provide further support for the ML analysis and reveal in greater detail, and therefore gain further insights into the phylogeny of *R. appendiculatus*, median-joining (MJ) network [46] was constructed using COI sequences with NETWORK 4.6 software (fluxus-engineering.com).

## Results

### Confirmation of the species identification

The 332 samples used in this study generated 558 bp of high quality consensus COI sequences. Their molecular identity was confirmed via BLASTN searches against the NCBI’s non-redundant nucleotide sequence database. The BLASTN searches returned high values of sequence similarity (97–100 %) with those of archived *R. appendiculatus* (GenBank AF132833; KC503257 and DQ859261).

### COI sequence diversity

The 558 bp fragment of COI revealed 30 polymorphic sites which defined 28 haplotypes (Additional file 3: Table S3). All of the 28 haplotypes were deposited in the GenBank database under accession numbers KU725890-KU725917 while the protein identifiers for the corresponding translated protein sequences for these haplotypes were ANF89378-ANF89405. Two haplotypes (Hap_4 and Hap_1) were defined by 107 (32.2 %) and 94 (28.3 %) sequences, respectively, accounting for 60.5 % of all sequences analysed (Additional file 3: Table S3). Hap_4 was exclusive to Muguga low-line (ML), Lab OlPejeta (LP) and Zambia Eastern Province (ZE) populations and was observed in eight out of the 12 sequences from Kiambu High-line (KH). Hap_1 was exclusive to South Africa Lab (SAL), South Africa Natal (SAN), Kiambu unselected line (KU) and Zimbabwe West Mashonaland (ZM) and was also observed in eight of the 12 sequences from Muguga infected (MF) and Zambia Southern Province (ZS) and in 19 out of the 20 sequences from Kilifi (KF) (Table 2). All sequences from Uganda (UG) (*n* = 12) were exclusively of one haplotype (Hap_8). The average number of haplotypes across the 22 study populations was 28 and on average, 20, 14, 7 and 5 haplotypes were observed in tick populations sampled from areas grazed by cattle only, co-grazed by cattle and wildlife, grazed by wildlife only and in laboratory stocks, respectively (Table 1). The highest number of haplotypes (ten) was observed in Kitale (KT) and Field OlPejeta (FP) while the lowest (one) was observed in nine laboratory stocks. Haplotype sequences of each of the 22 studied populations were deposited in GenBank under the Accession numbers KX276862-KX276944 (Table 1).

**Table 2:** Distribution of tick samples from different populations in the four major haplotypes

Haplogroup	Haplogroup A		Haplogroup B		
	Subgroup I	Subgroup II			
Population Name	Number of sequences in Hap_4	Number of sequences in Hap_5	Number of sequences in Hap_1	Number of sequences in Hap_7	Total sample size^a^
NB	8	8	–	1	21
KT	8	6	4	1	29
ML	11	–	–	–	11
MU	6	–	1	–	12
MF	2	–	8	–	12
MA	7	2	1	4	22
RUM2	1	1	2	1	5
LP	14	–	–	–	14
FP	9	3	–	1	23
BO	11	–	–	–	23
KH	8	–	4	–	12
KU	–	–	10	–	10
MK	9	6	1	–	25
ZE	12	–	–	–	12
RU	1	–	6	10	21
ZS	–	–	8	–	8
SAL	–	–	5	–	5
SAN	–	–	5	–	5
ZM	–	–	12	–	12
BU	–	2	8	3	18
KF	–	–	19	–	20
Total	107	28	94	21	

^a^Total number sequences from each of the populations that were analysed

(−) indicates that no samples from that particular population were included

Only haplotypes represented by more than 20 sequences are shown

The haplotype diversity ranged from 0.900 ± 0.161 (mean ± standard deviation) in Ruma (RUM2) to 0 in nine laboratory stocks with an average value of 0.802 ± 0.014 (Table 1). Amongst tick populations sampled from the areas grazed by different host species, those from areas grazed exclusively by wildlife had the highest haplotype diversity (mean 0.767 ± 0.0064) and the laboratory stocks had the lowest (mean 0.143 ± 0.029). The average nucleotide diversity was 0.0123 ± 0.00019 ranging from 0 in nine laboratory stocks to 0.010 ± 0.06 in Kitale (KT) (Table 1). The average number of nucleotide differences was 6.865 ± 3.2391 and ranged from 0 in nine laboratory stocks to 8.100 ± 4.534 in RM (Table 1). In general, ticks from field populations showed the highest levels of diversity whereas the laboratory stocks were the least diverse.

### Phylogenetic relationships and median-joining network of COI haplotypes

**Fig. 1:**
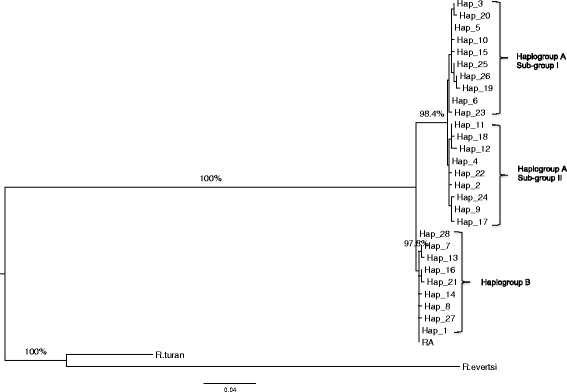
Phylogenetic tree showing the relationships between the 28 *Rhipicephalus appendiculatus* COI haplotypes and a reference sequence from GenBank (AF132833 [RA]). The 28 haplotypes are represented by Hap 1–28. Percent bootstrap values above 75 % (1000 replications) are shown. COI sequence of *R. turanicus* (JQ737086) from the GenBank database and another from a Kenya tick confirmed to be *Rhipicephalus evertsi* were included as the outgroup

**Fig. 2:**
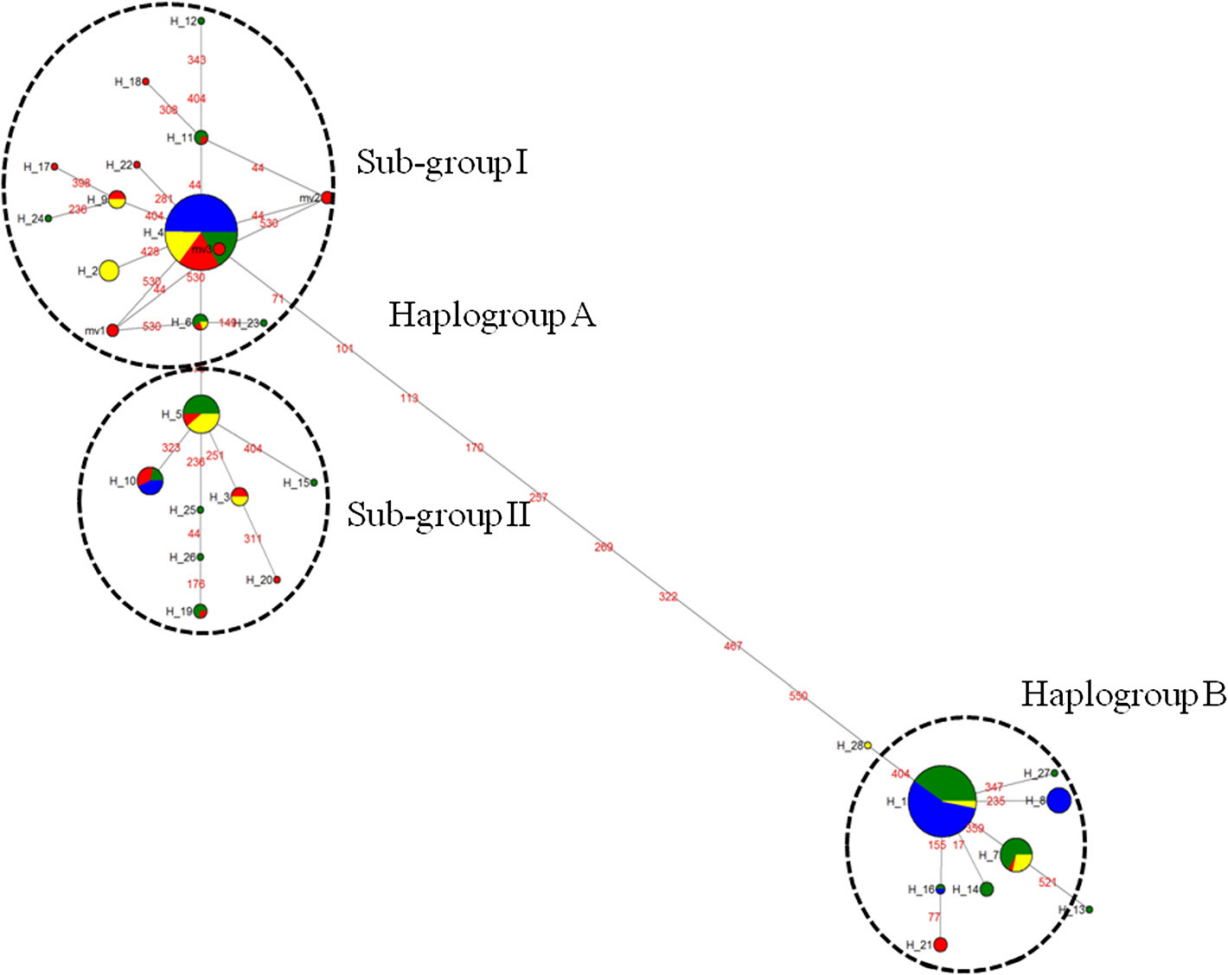
Median-Joining network of 28 COI haplotypes observed in 332 *Rhipicephalus appendiculatus* ticks. The network was based on the polymorphic sites in the 558 bp COI gene segment. Each circle represents a haplotype and the area of the circle is proportional to the haplotype frequency. Numbers represent nucleotide position. Colours represent a group of tick populations classified on the basis of the origin of the sequences: *blue*, laboratory stocks; *yellow*, populations sampled from pastures grazed by wildlife; *red*, populations sampled from pastures grazed by both cattle and wildlife*; green*, populations sampled from cattle pastures. Median vectors are represented by “mv”

To gain insights into the phylogenetic relationships between the 28 COI haplotypes, a ML tree (Fig. 1) and a MJ network were constructed (Fig. 2). The ML tree revealed two well-resolved groups of *R. appendiculatus* (bootstrap value of 100 %). The MJ network also revealed two groups that were separated by 12 mutation steps. The cluster of haplotypes in the ML tree and MJ network did not differ between the two algorithms. We therefore designated the two groups as haplogroups A and B, respectively. Haplogroup A clustered 19 haplotypes including Hap_4, the haplotype with the highest frequency, whereas haplogroup B contained nine haplotypes, which included Hap_1, the haplotype with the second highest frequency. Two median vectors (mv) were observed among the two haplogroups (Fig. 2); they may represent either haplotypes that were not sampled, or alternatively never present in Kenya, or have become extinct. A star-like pattern anchored by haplotypes H_4 and H_1 was evident for haplogroup A and B (Fig. 2), respectively, hinting at population expansion from an ancestral group, although the timescale is unclear. The ML tree (Fig. 1) appears to suggest the presence of two sub-haplogroups within haplogroup A (bootstrap value of 98 %). These can also be observed within the MJ network but are separated by a single mutation step. This suggests the possibility of genetic divergence within haplogroup A, requiring further analysis using a larger set of samples.

**Fig. 3:**
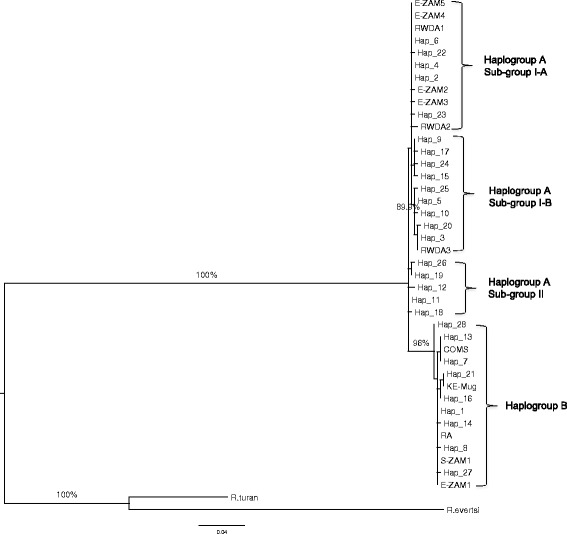
Tree showing the phylogenetic relationships between the Kenyan COI haplotypes and sequences generated by Mtambo et al. [17]. Eleven sequences from GenBank were included in the analysis. Five were from eastern Zambia [accession number DQ859261 (E-ZAM1); DQ859263 (E-ZAM2); DQ859264 (E-ZAM3); DQ859265 (E-ZAM4) and DQ859266 (E-ZAM5)], one from southern Zambia [DQ859262 (S-ZAM1)], three from Rwanda [DQ901360 (RWDA1), DQ901362 (RWDA2), DQ901363 (RWDA3)], one from Comoros Island [DQ901357 (COMS)] and one from Kenya [DQ901358 (KE-Mug)]. Another *R. appendiculatus* sequence [AF132833 (RA)] was included in the analysis as a reference while a sequence from *R. turanicus* [JQ737086 (*R. turan*)] was used as the outgroup. Percent bootstrap values above 75 % are shown

To test if the COI haplotypes generated in our study clustered with those of *R. appendiculatus* populations from eastern and southern Africa, which were also separated into two distinct groups [17], we reconstructed a ML tree using a 415 bp region derived from our 28 haplotypes combined with ten haplotypes defined by Mtambo et al. [17]. We used a 415 bp fragment because this was the size of the fragment amplified by Mtambo et al. [17]. Our haplotypes of haplogroup A clustered together with representative haplotypes from Zambia’s eastern province and Rwanda whereas those of haplogroup B clustered together with representative haplotypes from the Comoro Islands and one haplotype each from Zambia’s southern and eastern provinces, respectively (Fig. 3). Further examination of this tree reveals that the haplotypes that formed haplogroup A were subdivided into three sub-haplogroups (bootstrap values > 89 %) (Fig. 3). One sub-haplogroup (sub-haplogroup II) contained Kenyan haplotypes only (*n* = 5), another (sub-haplogroup I-B) comprised nine Kenyan haplotypes and one from Rwanda, while the third (sub-haplogroup I-A) was made up of five haplotypes from Kenya, two from Rwanda and four from Zambia’s eastern province. This result suggests higher variation in *R. appendiculatus*, especially in haplogroup A, and a higher degree of phylogenetic complexity in this haplogroup not revealed in the studies of Mtambo et al. [17, 18].

### Population structure and demographic dynamics deduced from COI sequences

The partition of genetic variation within and among populations and groups of populations was investigated using hierarchical AMOVA taking into account seven population groups defined *a priori* (Table 3). The highest level of genetic variation (90.8 %) was attributable to genetic differences between haplogroups A and B. Only 4.92 % of the total variation could be assigned to differences associated with the three host species complexes (cattle, cattle-wildlife and wildlife, respectively). Generally, the lowest levels of genetic variation were observed between groups of populations and ranged from−1.43 to 14.91 %. The variation present among individuals within populations ranged between 9.3 and 52.37 % whereas that among populations within groups was greater than 35 % in four comparisons. The observed variation between individuals within populations was greater than 43 % with the exception of the comparison amongst haplogroups (Table 3).

**Table 3:** Global analysis of molecular variance (AMOVA) for different groups of ticks at different hierarchical levels

Clusters	Hierarchy	Variance components	Percentage variation
Overall (all *R. appendiculatus* populations)	1	Among populations	56.82
		Within populations	43.18
Cattle *vs* cattle-wildlife and wildlife *R. appendiculatus* populations	2	Among groups	14.91
		Among populations within groups	35.38
		Within populations	49.71
Cattle *vs* wildlife only *R. appendiculatus* populations	2	Among groups	3.88
		Among populations within groups	47.82
		Within populations	48.30
Cattle-wildlife *vs* wildlife *R. appendiculatus* populations	2	Among groups	-4.96
		Among populations within groups	12.91
		Within populations	92.05
Field *vs* laboratory *R. appendiculatus* populations	2	Among groups	-1.43
		Among populations within groups	57.28
		Within populations	44.15
Cattle *vs* cattle-wildlife vs. wildlife *R. appendiculatus* populations	3	Among groups	4.94
		Among populations within groups	42.69
		Within populations	52.37
Haplogroup A *vs* haplogroup B (between the two major *R. appendiculatus* haplogroups)	2	Among populations	90.80
		Within populations	9.30

Clusters were based on *a priori* groupings of sampling localities. Cattle *R. appendiculatus* populations were collected directly from cattle or pastures grazed by cattle only. Cattle vs. cattle-wildlife and wildlife refers to populations collected from areas grazed by cattle versus a combination of populations from pastures co-grazed by cattle and wildlife and areas grazed by wildlife. Cattle *vs* wildlife only *R. appendiculatus* populations refer to ticks collected from areas grazed by cattle versus those collected areas grazed by wildlife only. Cattle-wildlife *vs* wildlife populations refer to populations from areas co-grazed by both cattle and wildlife versus wildlife only populations. Field *vs* laboratory *R. appendiculatus* populations refers to all *R. appendiculatus* ticks collected from field localities versus laboratory *R. appendiculatus*. Haplogroup A *vs* haplogroup B was between the two major *R. appendiculatus* haplogroups identified by ML and MJ network

**Table 4:** *R. appendiculatus* mismatch distribution analysis and selective neutrality test statistics for 332 tick samples and the two major *R. appendiculatus* haplogroups

Parameters	Overall	Haplogroup A	Haplogroup B
Sum of Squared deviation (SSD)	0.0761	0.0126	0.0028
*P* (Simulated SSD ≥ Observed SSD)	0.034	0.550	0.17
Harpending’s Raggedness index (RI)	0.061	0.05029	0.102
*P* (Simulated RI ≥ Observed RI)	0.060	0.740	0.210
Tajima’s D	1.244	-1.65063	-1.08714
Tajima’s D *P*-value	0.914	0.017	0.125
*F*_*S*_	-0.1222	-10.3479	-3.46287
*F*_*S*_ *P*-value	0.549	0.000	0.057

**Fig. 4:**
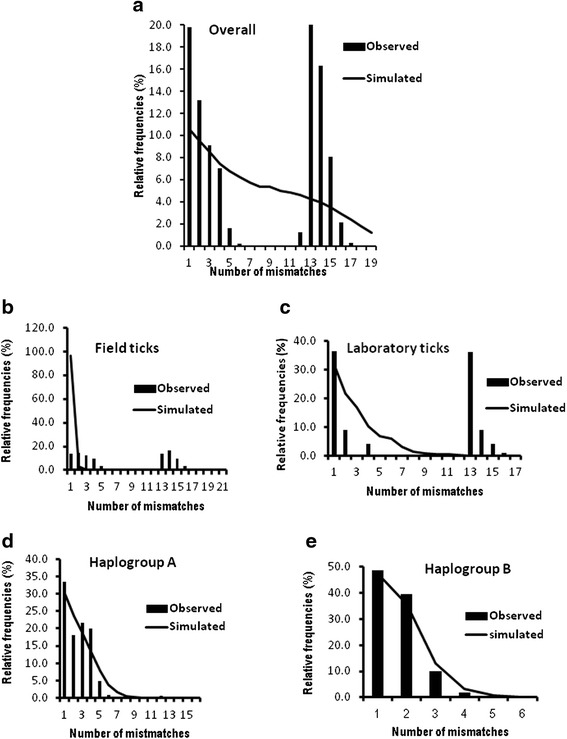
(**a**) shows the overall mismatch distribution pattern for the 22 *R*. *appendiculatus* populations analysed. (**b**) and (**c**) depict the distribution profiles of 10 field and 12 laboratory populations respectively. (**d**) and (**e**) shows the distribution patterns of ticks in haplogroup A and B respectively

To provide insight into demographic dynamics, we analysed mismatch distribution patterns for different groups of populations. The overall mismatch distribution pattern for the 22 populations (Fig. 4a) was bimodal. The observed pattern did not deviate significantly from that expected under a model of expansion (*SSD* = 0.076, *P* = 0.09) and had a smooth distribution (*RI* = 0.061, *P* = 0.070) (Table 1). The Tajima’s *D* statistic was positive while Fu’s *F*_S_ was negative and neither were significant (Table 1). Taken together, these data are consistent with population expansion. We also investigated the demographic profiles for the ten populations sampled from the field and the 12 laboratory stocks (Fig. 4b, c). Both groups of populations exhibited two peaks. The observed pattern for the field populations deviated significantly from the one expected under a model of expansion (*SSD* = 0.845, *P* < 0.0001) with no significant variation around the curve (*RI* = 0.0317, *P* = 1.000). For this group, Tajima’s *D* statistic was positive (*D* = 0.767) but this was not significant (*P* = 0.819), while Fu’s *F*_S_ parameter was negative (*F*_S_ = -0.959) and not significant (*P* = 0.447). For the laboratory stocks, the observed pattern also deviated significantly from that expected (*SSD* = 0.189, *P* = 0.04). For this group of ticks, both Tajima’s *D* statistic (*D* = 3.199, *P* = 0.996) and Fu’s *F*_S_ parameter (*F*_S_ = 15.042, *P* = 0.994) were positive but not significant. This suggests that either the field populations have a weak signal of expansion or are in demographic equilibrium, whereas the laboratory-bred stocks have been subject to an anthropogenic bottleneck and/or genetic drift. The two peaks observed in the overall dataset and in the field and laboratory stocks, respectively suggested the existence of two groups of ticks. The two peaks were found to correspond to the two haplogroups revealed by the ML and MJ analyses. We therefore performed mismatch analysis for each haplogroup (Fig. 4d, e). Both exhibited a unimodal profile and the observed patterns did not deviate significantly from that expected under a scenario of population expansion (Haplogroup A: *SSD* = 0.0126, *P* = 0.550; *RI* = 0.050, *P* = 0.740; haplogroup B: *SSD* = 0.0028, *P* = 0.170; *RI* = 0.102, *P* = 0.210). Both had negative Tajima’s *D* (-1.650 and -1.087, respectively) and Fu’s *F*_S_ (-10.348 and−3.462, respectively) values (Table 4). While Tajima’s *D* (*P* = 0.017) and Fu’s *F*_S_ (*P* <0.001) attained significance for haplogroup A, the values for haplogroup B did not (*P* = 0.125, *P* = 0.057) (Table 4). This suggests a strong signal of expansion for haplogroup A and a weaker one for haplogroup B. Our recent study utilizing nuclear satellite markers had also observed population expansion in field ticks [21]. These findings together with the star-like pattern observed in the MJ network, the mismatch distribution patterns and the two coalescent-based estimators of neutrality indicate expansion in the two haplogroups even in the absence of molecular dating.

### Diversity and phylogenetic relationships based on 12S rRNA and ITS2 region

The 12S rRNA gene and ITS2 region were sequenced from a subset of the 332 *R. appendiculatus* individuals sequenced for the COI gene. Of the 93 12S rDNA sequences from 12 populations, five haplotypes were observed, two main (one defined by 38 sequences and the other by 52 sequences, respectively), and three minor (each defined by one sequence). Following ML phylogeny analysis, the five haplotypes clustered into two haplogroups which were identical to those generated from the COI gene.

A 1149 bp fragment of the ITS2 region was amplified from 87 individuals derived from different mitochondrial haplotypes. Three haplotypes were observed. One contained 67 sequences and the other two contained nine and 11 sequences, respectively. These ITS sequences did not cluster into groups corresponding to the COI or 12S rDNA haplogroups. The five 12S rDNA haplotype sequences were deposited in the GenBank database under accession numbers KX276945-49 and those of the three ITS2 haplotypes unde accession numbers KX276950-52.

## Discussion

This study assessed the genetic relationships between populations of *R. appendiculatus* found in Kenya through the analysis of the mitochondrial COI and 12S rRNA genes and the nuclear transcribed ribosomal ITS2 fragment. COI gene has been and continues to be widely used as a marker for DNA barcoding to discriminate between closely related taxa [47–52]. Evolution of the COI gene is thought to be rapid enough to allow the discrimination of closely related species, as well as to detect intraspecific differentiation of phylogeographically distinct groups [53, 54]. The utility of COI as a phylogenetic marker for ticks has been demonstrated previously [55–58]. It has also been used previously to show *R. appendiculatus* speciation [17, 18, 59] and the current study found the variation in the COI to be adequate for phylogeny reconstruction and associated analyses using *R. appendiculatus* samples from Kenya.

In reconstructing the phylogenetic history of a species, the use of multiple genetic markers targeting different regions of the genome, is a better strategy in order to overcome the drawbacks of using a single marker, while increasing the accuracy of inference [60, 61]. Here in addition to the COI gene, we analysed the phylogenetic relationships using the mitochondrially-encoded 12S rRNA gene and the nuclear genome-encoded ITS2 fragment. The COI analysis identified 28 haplotypes in 332 sequences. The NJ and MJ network partitioned these haplotypes into two distinct haplogroups. These two haplogroups were also discriminated by the 12S rDNA sequences but not by the nuclear transcribed ITS2 sequences. Using COI and 12S rDNA, Mtambo et al. [17, 18] also observed two haplogroups of *R. appendiculatus* in eastern and southern Zambia but these were not detected by the ITS2 sequences. The low resolution afforded by ITS2 has also been reported in *Amblyomma hebraeum* and *Hyalomma rufipes* [6]. These findings suggest that COI and 12S rRNA genes are better markers for studying intraspecific diversity whereas the ITS2 fragment may be more useful in discriminating between species because it tends to show little intraspecific, but, considerable interspecific variation, possibly due to sexual recombination within species [62].

From the analysis of 332 COI sequences of *R. appendiculatus*, the overall mean number of nucleotide differences was 6.8647 ± 3.2391 and the mean haplotype and nucleotide diversities were 0.802 ± 0.014 and 0.0123 ± 0.0064, respectively. Cangi et al. [6], observed a lower level of haplotype and nucleotide diversities of 0.66 and 0.002, respectively, in *A. hebraeum*, an ixodid tick with a wider vertebrate host range, but a comparable level of haplotype and nucleotide diversity among isolates of 0.96 and 0.009, respectively, relative to the much more host-specialized *H. rufipes*. We also observed a high level of intra- and inter-population genetic diversity among the study populations. The values were much higher in the field ticks compared to the laboratory stocks, which were, by definition, subject to founder effects and population bottlenecks. The high diversity in field ticks is most probably the result of admixture between different geographic populations facilitated by the translocation of domestic animals either as trade items or through exchange following socio-cultural traditions. Indeed, no phylogeographic structure was revealed between the *R. appendiculatus* populations analysed in this study as revealed by either ML or MJ network analysis. In an earlier study, Kanduma et al. [21] observed no phylogeographic structure in field ticks that were analysed using autosomal micro- and minisatellite markers. The results suggest extensive translocation of ticks over a wide geographic range, in spite of low intrinsic dispersal ability of these arthropods resulting in populations with admixed genotypes. Domestic cattle in Kenya are frequently moved over large distances for commercial and socio-cultural reasons, as well as for seeking pasture during dry seasons. These would facilitate tick dispersal over a large geographical range, while the movement of the natural reservoirs of *R. appendiculatus* (wild bovidae) within the wildlife areas considered in this study is limited since these areas are fenced.

The laboratory stocks investigated here have been maintained as closed populations for over 30 years. It is therefore not surprising that they exhibited low levels of genetic diversity due to inevitably high levels of inbreeding. In spite of their inbred status, AMOVA revealed a negative value of genetic differentiation between the field and laboratory stocks implying that the two groups are much more related than might be expected. There are several potential explanations. First, that the inbreeding in the laboratory stocks has not resulted in a drastic reduction in their allelic variation; secondly, that variation present in the laboratory stocks is well represented in the field stocks; and thirdly, the induced bottleneck and genetic drift which could be due to inbreeding and small effective population sizes have not altered drastically their allelic composition.

Morphological [19], physiological [13] and phylogenetic [17, 18] data previously identified two distinct groups of field *R. appendiculatus* in some parts of Africa and it was suggested that they may represent geographically differentiated lineages, that may have diversified as a result of distinct selective pressures. For instance, ticks found in southern Africa (South Africa, southern Zambia and Zimbabwe) and those found in eastern Africa (Kenya, Tanzania, Uganda, Burundi and Rwanda) were thought to constitute two geographically isolated groups of ticks that can be discriminated based on morphological, ecological and epidemiological differences [17, 18]. In the current study, we observed two major haplogroups of *R. appendiculatus* in Kenya as defined by mitochondrial haplotype. These two haplogroups however exhibited no phylogeographic structure or correlation with the type of host species from which the ticks were collected or the evolutionary and breeding history of the species (field populations relative to laboratory stocks). Although we did not estimate the divergence time between the two genetic groups, it is possible that their divergence is not recent because they were observed among inbred laboratory stocks which were initially collected from populations of field ticks up to 50 years ago. AMOVA revealed that 90.8 % of the total genetic variation was explained by divergence within the two major haplogroups. While different host species have been shown to influence the spatio-genetic structure of other tick species, such as *Ixodes uriae* [5], the genetic variation between *R. appendiculatus* collected from different mammalian hosts was low (4.94 %). By contrast, the between population variation exceeded 35 %, whereas the variation between individuals within populations ranged between 9.3 and 52.3 %. This demonstrates low genetic differentiation between populations of *R. appendiculatus* sampled from different hosts suggesting minimal host specialisation. This suggests that genetic differentiation amongst tick populations in Kenya is a phenomenon primarily of ancestral differentiation between the two haplogroups and that recent reproductive isolation and the exploitation of different mammalian hosts has, to date, played a relatively minor role in driving this differentiation. The fact that the two major haplogroups that we have identified clusters together with representative haplotypes of *R. appendiculatus* from southern Africa [17, 18], suggests a wide geographic distribution range of these haplogroups in eastern and southern Africa. It is possible that the original divergence in this species could have arisen either due to genetic drift and/or novel adaptations via selection giving rise to significant morphological, physiological and phenotypic changes seen in ticks from different geographical areas. Whether there are any associated phenotypic differences that can be used to discriminate the two haplogroups, which might influence parameters such as *T. parva* transmission dynamics, requires further investigation. Further investigation is also required into origin and possible evolutionary forces driving the occurrence of multiple sub-haplogroups within haplogroup A.

We investigated the demographic dynamics of *R. appendiculatus* in Kenya by assessing the mismatch distribution patterns for the overall dataset, the field and laboratory stocks and within the two haplogroups identified by the ML and MJ network analysis. The results for the overall dataset, field populations and the two haplogroups suggest that these three groups of *R. appendiculatus* have passed through a demographic expansion perhaps associated with range expansion of a founder population.

The findings of this study may have taxonomic implications and suggest the potential for incipient speciation in *R. appendiculatus. Rhipicephalus appendiculatus* is a generalist tick, although Cape buffalo is the main wild host reservoir, and cattle are the preferred domestic hosts of the adult and nymphal instars [63, 64]. Such a generalist ectoparasite which infests other wild and domestic animals can disperse across ecosystems potentially modifying disease transmission cycles. In this respect, understanding the population structure of *R. appendiculatus* is important in the design of sustainable control strategies, since different tick populations may be characterised by differences in vector competence, acaricide resistance and susceptibility to infection with *T. parva*. In future it will be important to establish how the phenotypes of the two *R. appendiculatus* haplogoups identified in this study differ, particularly with respect to acquisition and transmission of ECF.

## Conclusions

COI and 12S genes are superior genetic markers for intra-species population genetic studies in *R. appendiculatus* over the ITS2. Based on these two genes, two distinct and well-differentiated haplogroups which have passed through a demographic expansion perhaps associated with range expansion of a founder population exist in Kenya. These two haplogroups have no phylogeographic structure or correlation with their mammalian host species or the evolutionary and breeding history of the species. There is a wide geographical distribution range of these two haplogroups in eastern and southern Africa. These findings may have important taxonomic implications and may point to an ongoing speciation of *R. appendiculatus* in sub-Saharan Africa. It would be important to establish if the two haplogroups have any associated phenotypic differences which might influence parameters such as *T. parva* acquisition and transmission dynamics. In addition, identifying evolutionary forces driving the observed genetic differentiation may help explain the apparent population expansion of the two haplogroups within the sub-Saharan region.

## Abbreviations

AMOVA, analysis of molecular variance; ARC-OVI, Agricultural Research centre- Onderstepoort Veterinary Institute; AusAID, Australian Agency for International Development; AWARD, African Women in Agricultural Research and Development; BecA, Biosciences eastern and central Africa; BLASTN, basic local alignment search tool; bp, base pair; CIDA, Canadian International Development Agency; COI, cytochrome *c* oxidase subunit I; CSIRO, Australia’s Commonwealth Scientific and Industrial Research Organisation; DAAD, German academic exchange service; DNA, deoxyribonucleic acid; dNTP, deoxyribonucletide triphosphate; dNTPs, deoxyribonucleotide triphosphates; ECF, east coast fever; ICARDA, International Centre for Agricultural Research in the Dry Areas; *icipe*, International Centre of Insect Physiology and Ecology; ILRI, International Livestock Research Institute; ITS2, Internal transcribed spacer 2; KWS, Kenya Wildlife Service; MJ, median joining; ML, maximum likelihood; mv, median vector; NCBI, National Center Bioinformatics; NEPAD, new partnership for Africa’s development; PCR, polymerase chain reaction; rDNA, ribosomal deoxyribonucleic acid; SFSA, Syngenta Foundation for Sustainable Agriculture; SSD, sum of squares deviation

## Additional files

Additional file 1: Table S1. Number of ITS2 and 12S rDNA sequences analysed from eight field and four laboratory populations of *R. appendiculatus.* (DOCX 14 kb) Additional file 2: Table S2. PCR primer sequences for each gene marker amplified and their corresponding annealing temperatures. (DOCX 15 kb) Additional file 3: Table S3.
*R. appendiculatus* COI haplotypes showing variable sites and the number sequences in each haplotype. (DOCX 20 kb)
