# Supplementary material for: Analyses of mitochondrial genes reveal two sympatric but genetically divergent lineages of Rhipicephalus appendiculatus in Kenya
Source: Parasit Vectors. 2016 Jun 22;9:353. doi: 10.1186/s13071-016-1631-1 (PMC4918217; doi:10.1186/s13071-016-1631-1)
Supplement: Additional file 1: Table S1. — Number of ITS2 and 12S rDNA sequences analysed from eight field and four laboratory populations of R. appendiculatus. (DOCX 14 kb) [file 13071_2016_1631_MOESM1_ESM.docx]

Supplementary Table S1: Number of ITS2 and 12S sequences analysed from eight field and four laboratory populations of *R. appendiculatus*

| ***R. appendiculatus* population** | **No. of sequences** | |
| --- | --- | --- |
|  | **12S rDNA** | **ITS2** |
| BU | 5 | 9 |
| BO | 10 | 13 |
| FP | 6 | 7 |
| KF | 4 | 8 |
| MK | 13 | 4 |
| MF | 8 | 7 |
| KU | 7 | 9 |
| KT | 12 | 7 |
| ML | 8 | 7 |
| MU | 6 | 5 |
| RU | 9 | 11 |
| RUM2 | 5 | 0 |
| **Total** | **93** | **87** |
