# Supplementary material for: Analyses of mitochondrial genes reveal two sympatric but genetically divergent lineages of Rhipicephalus appendiculatus in Kenya
Source: Parasit Vectors. 2016 Jun 22;9:353. doi: 10.1186/s13071-016-1631-1 (PMC4918217; doi:10.1186/s13071-016-1631-1)
Supplement: Additional file 2: Table S2. — PCR primer sequences for each gene marker amplified and their corresponding annealing temperatures. (DOCX 15 kb) [file 13071_2016_1631_MOESM2_ESM.docx]

| Type of primer | Name of primer and its nucleotide sequence | Annealing temperature | Reference |
| --- | --- | --- | --- |
| COI forward | LCO1490  5′-GGTCAACAAATCATAAAGATATTGG-3′ | 40°C | [33] |
| COI reverse | HC02198  5′-TAAACTTCAGGGTGACCAAAAAATCA-3′ |  |  |
| 12S rDNA  forward | SR-J-1499  5′-TACTATGTTACGACTTAT-3′ | 50°C | [34] |
| 12S rDNA  reverse | SR-N-14594  5′-AAACTAGGATTAGATACCC-3′ |  |  |
| ITS2 forward  (full-length fragment) | 3SAF  5′-CTAAGCGGTGGATCACTCGG-3′ | 55°C | [35] |
| ITS2 reverse (full-length fragment ) | ITS2R  5′-ATATGCTTAAATTCAGCGGG-3′ |  | [36] |
| ITS2 forward  (inner fragment) | ITS2_Int_F1  5′-AAGAGCCTGCAGGGAAAG-3′ | 52°C |  |
| ITS2 reverse (inner fragment) | ITS2_Int_R1  5′-CACGTTCGTAAACCCATC-3′ |  |  |

Supplementary Table S2: PCR primer sequences for each gene marker amplified and their corresponding annealing temperatures
