# Supplementary material for: Analyses of mitochondrial genes reveal two sympatric but genetically divergent lineages of Rhipicephalus appendiculatus in Kenya
Source: Parasit Vectors. 2016 Jun 22;9:353. doi: 10.1186/s13071-016-1631-1 (PMC4918217; doi:10.1186/s13071-016-1631-1)
Supplement: Additional file 3: Table S3. — R. appendiculatus COI haplotypes showing variable sites and the number sequences in each haplotype. (DOCX 20 kb) [file 13071_2016_1631_MOESM3_ESM.docx]

Supplementary Table S3: *R. appendiculatus* COI haplotypes showing variable sites and the number sequences in each haplotype

| **POSITIONª** | **17** | **44** | **71** | **77** | **101** | **113** | **149** | **155** | **170** | **176** | **235** | **236** | **251** | **257** | **269** | **281** | **308** | **311** | **322** | **323** | **343** | **347** | **359** | **398** | **404** | **428** | **467** | **521** | **530** | **550** | **N⃰** |
| --- | --- | --- | --- | --- | --- | --- | --- | --- | --- | --- | --- | --- | --- | --- | --- | --- | --- | --- | --- | --- | --- | --- | --- | --- | --- | --- | --- | --- | --- | --- | --- |
| **Hap_1** | T | A | C | A | A | T | G | A | G | C | G | C | A | G | A | T | A | A | G | T | G | T | A | T | A | T | A | T | A | A | 94 |
| **Hap_2** | **.** | C | T | **.** | G | C | **.** | **.** | A | **.** | **.** | **.** | **.** | A | G | **.** | **.** | **.** | A | **.** | **.** | **.** | **.** | **.** | G | C | G | **.** | C | G | 8 |
| **Hap_3** | **.** | C | T | **.** | G | C | **.** | **.** | A | T | **.** | **.** | G | A | G | **.** | **.** | **.** | A | **.** | **.** | **.** | **.** | **.** | G | **.** | G | **.** | T | G | 6 |
| **Hap_4** | **.** | C | T | **.** | G | C | **.** | **.** | A | **.** | **.** | **.** | **.** | A | G | **.** | **.** | **.** | A | **.** | **.** | **.** | **.** | **.** | G | **.** | G | **.** | C | G | 107 |
| **Hap_5** | **.** | C | T | **.** | G | C | **.** | **.** | A | T | **.** | **.** | **.** | A | G | **.** | **.** | **.** | A | **.** | **.** | **.** | **.** | **.** | G | **.** | G | **.** | T | G | 28 |
| **Hap_6** | **.** | C | T | **.** | G | C | **.** | **.** | A | **.** | **.** | **.** | **.** | A | G | **.** | **.** | **.** | A | **.** | **.** | **.** | **.** | **.** | G | **.** | G | **.** | T | G | 5 |
| **Hap_7** | **.** | **.** | **.** | **.** | **.** | **.** | **.** | **.** | **.** | **.** | **.** | **.** | **.** | **.** | **.** | **.** | **.** | **.** | **.** | **.** | **.** | **.** | G | **.** | **.** | **.** | **.** | **.** | **.** | **.** | 21 |
| **Hap_8** | **.** | **.** | **.** | **.** | **.** | **.** | **.** | **.** | **.** | **.** | A | **.** | **.** | **.** | **.** | **.** | **.** | **.** | **.** | **.** | **.** | **.** | **.** | **.** | **.** | **.** | **.** | **.** | **.** | **.** | 12 |
| **Hap_9** | **.** | C | T | **.** | G | C | **.** | **.** | A | **.** | **.** | **.** | **.** | A | G | **.** | **.** | **.** | A | **.** | **.** | **.** | **.** | **.** | **.** | **.** | G | **.** | C | G | 6 |
| **Hap_10** | **.** | C | T | **.** | G | C | **.** | **.** | A | T | **.** | **.** | **.** | A | G | **.** | **.** | **.** | A | C | **.** | **.** | **.** | **.** | G | **.** | G | **.** | T | G | 14 |
| **Hap_11** | **.** | T | T | **.** | G | C | **.** | **.** | A | **.** | **.** | **.** | **.** | A | G | **.** | **.** | **.** | A | **.** | **.** | **.** | **.** | **.** | G | **.** | G | **.** | C | G | 4 |
| **Hap_12** | **.** | T | T | **.** | G | C | **.** | **.** | A | **.** | **.** | **.** | **.** | A | G | **.** | **.** | **.** | A | **.** | A | **.** | **.** | **.** | T | **.** | G | **.** | C | G | 1 |
| **Hap_13** | **.** | **.** | **.** | **.** | **.** | **.** | **.** | **.** | **.** | **.** | **.** | **.** | **.** | **.** | **.** | **.** | **.** | **.** | **.** | **.** | **.** | **.** | G | **.** | **.** | **.** | **.** | C | **.** | **.** | 1 |
| **Hap_14** | G | **.** | **.** | **.** | **.** | **.** | **.** | **.** | **.** | **.** | **.** | **.** | **.** | **.** | **.** | **.** | **.** | **.** | **.** | **.** | **.** | **.** | **.** | **.** | **.** | **.** | **.** | **.** | **.** | **.** | 4 |
| **Hap_15** | **.** | C | T | **.** | G | C | **.** | **.** | A | T | **.** | **.** | **.** | A | G | **.** | **.** | **.** | A | **.** | **.** | **.** | **.** | **.** | **.** | **.** | G | **.** | T | G | 1 |
| **Hap_16** | **.** | **.** | **.** | **.** | **.** | **.** | **.** | G | **.** | **.** | **.** | **.** | **.** | **.** | **.** | **.** | **.** | **.** | **.** | **.** | **.** | **.** | **.** | **.** | **.** | **.** | **.** | **.** | **.** | **.** | 2 |
| **Hap_17** | **.** | C | T | **.** | G | C | **.** | **.** | A | **.** | **.** | **.** | **.** | A | G | **.** | **.** | **.** | A | **.** | **.** | **.** | **.** | C | **.** | **.** | G | **.** | C | G | 1 |
| **Hap_18** | **.** | T | T | **.** | G | C | **.** | **.** | A | **.** | **.** | **.** | **.** | A | G | **.** | G | **.** | A | **.** | **.** | **.** | **.** | **.** | G | **.** | G | **.** | C | G | 1 |
| **Hap_19** | **.** | T | T | **.** | G | C | **.** | **.** | A | **.** | **.** | T | **.** | A | G | **.** | **.** | **.** | A | **.** | **.** | **.** | **.** | **.** | G | **.** | G | **.** | T | G | 4 |
| **Hap_20** | **.** | C | T | **.** | G | C | **.** | **.** | A | T | **.** | **.** | G | A | G | **.** | **.** | G | A | **.** | **.** | **.** | **.** | **.** | G | **.** | G | **.** | T | G | 1 |
| **Hap_21** | **.** | **.** | **.** | G | **.** | **.** | **.** | G | **.** | **.** | **.** | **.** | **.** | **.** | **.** | **.** | **.** | **.** | **.** | **.** | **.** | **.** | **.** | **.** | **.** | **.** | **.** | **.** | **.** | **.** | 4 |
| **Hap_22** | **.** | C | T | **.** | G | C | **.** | **.** | A | **.** | **.** | **.** | **.** | A | G | C | **.** | **.** | A | **.** | **.** | **.** | **.** | **.** | G | **.** | G | **.** | C | G | 1 |
| **Hap_23** | **.** | C | T | **.** | G | C | T | **.** | A | **.** | **.** | **.** | **.** | A | G | **.** | **.** | **.** | A | **.** | **.** | **.** | **.** | **.** | G | **.** | G | **.** | T | G | 1 |
| **Hap_24** | **.** | C | T | **.** | G | C | **.** | **.** | A | **.** | **.** | T | **.** | A | G | **.** | **.** | **.** | A | **.** | **.** | **.** | **.** | **.** | **.** | **.** | G | **.** | C | G | 1 |
| **Hap_25** | **.** | C | T | **.** | G | C | **.** | **.** | A | T | **.** | T | **.** | A | G | **.** | **.** | **.** | A | **.** | **.** | **.** | **.** | **.** | G | **.** | G | **.** | T | G | 1 |
| **Hap_26** | **.** | T | T | **.** | G | C | **.** | **.** | A | T | **.** | T | **.** | A | G | **.** | **.** | **.** | A | **.** | **.** | **.** | **.** | **.** | G | **.** | G | **.** | T | G | 1 |
| **Hap_27** | **.** | **.** | **.** | **.** | **.** | **.** | **.** | **.** | **.** | **.** | **.** | **.** | **.** | **.** | **.** | **.** | **.** | **.** | **.** | **.** | **.** | C | **.** | **.** | **.** | **.** | **.** | **.** | **.** | **.** | 1 |
| **Hap_28** | **.** | **.** | **.** | **.** | **.** | **.** | **.** | **.** | **.** | **.** | **.** | **.** | **.** | **.** | **.** | **.** | **.** | **.** | **.** | **.** | **.** | **.** | **.** | **.** | G | **.** | **.** | **.** | **.** | **.** | 1 |

Sequence variation of 28 COI haplotypes derived from 332 *R****.*** *appendiculatus* ticks**.** There were a total of 30 polymorphic sites**.**

ª**Position**: Nucleotide position where variation occurred**.**

⃰N: number of individuals sharing the same haplotype**.**

Dots (**.**) Identity between sequences
